# Systemic Inflammation Impairs Mood Function by Disrupting the Resting-State Functional Network in a Rat Animal Model Induced by Lipopolysaccharide Challenge

**DOI:** 10.1155/2019/6212934

**Published:** 2019-05-09

**Authors:** Xia Zhu, Mu-Huo Ji, Shu-Ming Li, Bin Li, Li Mei, Jian-Jun Yang

**Affiliations:** ^1^Department of Anesthesiology, Jinling Clinical Medical College of Nanjing Medical University, Nanjing, Jiangsu, China; ^2^Department of Anesthesiology, The Affiliated Lianyungang Oriental Hospital of Kangda College of Nanjing Medical University, Lianyungang, Jiangsu, China; ^3^Department of Anesthesiology, Zhongda Hospital, Medical School, Southeast University, Nanjing, Jiangsu, China; ^4^Department of Anesthesiology, Guangdong General Hospital, Guangdong Academy of Medical Sciences, Guangzhou, China; ^5^Department of Anesthesiology, The First Affiliated Hospital of Zhengzhou University, Zhengzhou, Henan, China

## Abstract

**Background:**

Systemic inflammation impairs cognitive performance, yet the brain networks mediating this process remain to be elucidated. The purpose of the current study was to use resting-state functional magnetic resonance imaging (fMRI) to explore changes in the functional connectivity in a lipopolysaccharide- (LPS-) induced systemic inflammation animal model.

**Materials and Methods:**

We used the regional homogeneity (ReHo) method to examine abnormal brain regions between the control and LPS groups and then considered them as seeds of functional connectivity analysis.

**Results:**

Compared with the control group, our study showed that (1) LPS impaired mood function, as reflected by a depression-like behavior in the forced swim test; (2) LPS induced significantly increased ReHo values in the anterior cingulate cortex (ACC) and caudate putamen (CPu); (3) the ACC seed showed increased functional connectivity with the retrosplenial cortex, superior colliculus, and inferior colliculus; and (4) the right CPu seed showed increased functional connectivity with the left CPu. Linear regression analysis showed a LPS-induced depression-like behavior which was associated with increased ReHo values in the ACC and right CPu. Moreover, the LPS-induced depression-like behavior was related to increased functional connectivity between the right CPu and left CPu.

**Conclusion:**

This is the first study to show that systemic inflammation impairs mood function that is associated with an altered resting-state functional network based on ReHo analysis, providing evidence of the abnormal regional brain spontaneous activity which might be involved in inflammation-related neurobehavioral abnormalities.

## 1. Introduction

Sepsis-associated encephalopathy (SAE) is a central nervous system (CNS) complication induced by systemic inflammation in response to bacterial lipopolysaccharide (LPS) or other endotoxic bacterial cell wall components without direct brain infection [1–3]. Of note, SAE can affect up to 70% of patients with severe sepsis, leading to long-term neurobehavioral abnormalities, poor quality of life, and even increased mortality [2]. However, the pathophysiology of SAE remains largely to be elucidated [4–6]. Given the relevance of a disturbed neural circuit in the pathophysiology of many neuropsychiatric disorders [7–9], understanding the neural basis of inflammation-mediated affective and cognitive impairment is essential for its diagnosis and treatment.

Functional magnetic resonance imaging (fMRI) based on blood oxygen level–dependent (BOLD) contrast is a useful technique that has been extensively applied to study the brain and its functional organization in both healthy and disease states [7–9]. Although resting-state fMRI reflects spontaneous neuronal activity and functional connectivity indirectly, it requires no stimulus-response sequence and is highly replicable and thus receives increased attention among researchers investigating cognitively compromised patients [10] and depression [11]. Studies using fMRI have consistently found that neural networks within and between brain structures facilitate some functional purpose or neuronal processing [7, 8, 12]. On the contrary, disrupted or altered resting-state brain activities are frequently observed in Alzheimer's disease [13] and depression [14] and in patients following chemotherapy [15]. Strikingly, previous neuroimaging studies on sickness behavior have demonstrated that systemic inflammation alters the functional connectivity of brain networks within the human brain at rest [16, 17]. In these studies, one main approach being applied to characterize the resting-state networks is the seed-based functional connectivity in a priori defined subnetworks [16]. However, this method explored the fMRI signal from the aspect of temporal correlation but not regional brain spontaneous activity. On the other hand, regional homogeneity (ReHo) measures the functional coherence of a given voxel with its nearest neighbors [18], which can reflect the temporal homogeneity of neural activity and effectively evaluate resting-state brain activity. Currently, ReHo is a potentially powerful tool for investigating the alterations in resting-state brain activity, thereby complementing the information provided by functional connectivity analysis. Indeed, the ReHo algorithm is proven to be useful in detecting the changes in a variety of diseases such as major depression [19], Parkinson's disease [20], and schizophrenia [21]. As the BOLD signal of fMRI may reflect neural activity, abnormal ReHo is possibly relevant to the changes of temporal aspects of neural activity in the regional brain [18]. However, there is a paucity of information available on brain functional connectivity changes after peripheral LPS challenge based on ReHo analysis.

In light of these findings, we used the ReHo method to examine abnormal brain regions between the control and LPS groups. Regions showing ReHo differences were selected as seeds for further functional connectivity analysis. We hypothesized that LPS-induced mood and cognitive impairment involves altered ReHo values and neural connectivity when compared with the control group.

## 2. Materials and Methods

### 2.1. Animals and Study Protocol

**Figure 1:**
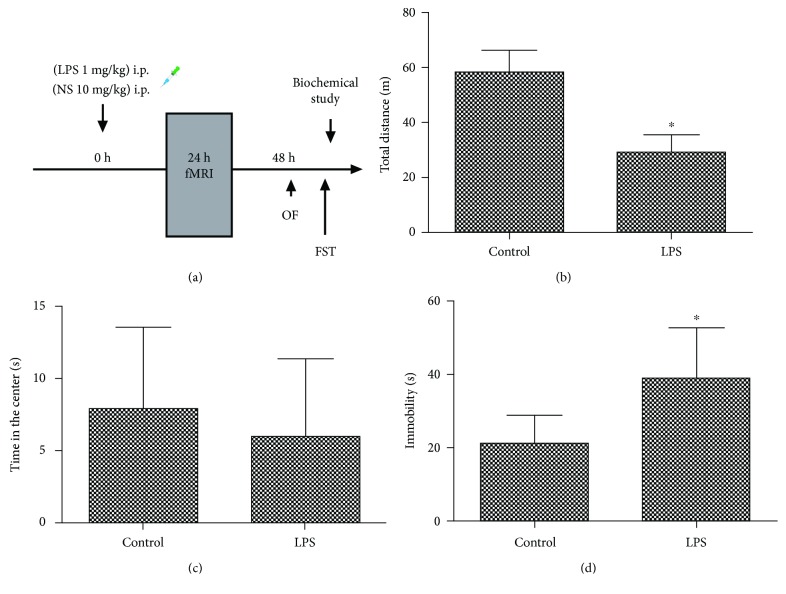
(a) Experimental design of the study. (b) Effects of LPS on total distance traveled in the open field. (c) Effects of LPS on time spent in the center in the open arena. (d) Effects of LPS on immobility time in the forced swimming test. OF: open field; FST: forced swim test. NS: normal saline; LPS: lipopolysaccharide. ^∗^*P* < 0.05 vs. control group.

Approval was issued by the Ethics Committee of Zhongda Hospital (Nanjing, China). All the experimental procedures were implemented under the Guide for the Care and Use of Laboratory Animals from the National Institutes of Health (Bethesda, MD, USA). As subjects, thirty-two male Sprague-Dawley rats weighing 320–380 g were purchased from the Animal Center of Jinling Hospital (Nanjing, China) and cultivated in 12 h light–dark cycles (lighting at 07:00, a.m.) at 24 ± 1°C, with free access to water and chow. One rat in the control group was excluded due to obvious head movement, and then fifteen rats in the control group and sixteen rats in the LPS group were included in the final analysis. The diagram of the study protocol is shown in Figure 1(a).

### 2.2. Systemic LPS Administration and Dosage

In the present study, the rats were injected with either LPS (Escherichia coli endotoxin 0111: B4, Sigma, Lot # 064M4125V, Shanghai, China; 1 mg/kg LPS, a single-dose, intraperitoneal injection) or equal-volume (0.9%) normal saline (a single-dose, intraperitoneal injection). To maintain circadian effects, all the injections were completed between 8:00 and 9:00 a.m. The dose of LPS was designed as neurobehavioral-abnormality-causing, but not lethal [22–24].

### 2.3. Resting-State fMRI Acquisition

MRI was examined by the 7.0 T Bruker PharmaScan MRI scanner (70/16 PharmaScan, Bruker BioSpin GmbH, Germany). A quadrature volume resonator (inner diameter 72 mm) was used for radio frequency transmission, and a 4-element surface coil array was used for signal reception. For anesthesia induction, a mixture of isoflurane (5% for induction and 0.3%-0.5% for maintenance) and dexmedetomidine sedation was used [11]. A nose mask with a bite bar was used to deliver the isoflurane mixture on the MRI bed and to fix the animal in prone position. During the MRI scan, the rat was prostrated on a custom-made holder to minimize head motion, while the respiration rate was controlled between 60 and 80 breaths/min. An echo-planar imaging (EPI) sequence was performed: matrix size = 64 × 64, flip angle = 30°, resolution = 0.5 mm × 0.5 mm, slice thickness = 1.0 mm, slice gap = 0, repetition time (TR) = 2 s, echo time (TE) = 18 ms, and volume = 180. A coplanar T2-weighted scan was also acquired.

### 2.4. ReHo Calculation and Seed-Based Functional Connectivity Analysis

Preprocessing and analysis of the fMRI data were performed using SPM8 (Functional Imaging Laboratory, Wellcome Department of Cognitive Neurology, London, UK). The ReHo calculation procedure was applied as previously described [18]. This is accomplished on a voxel-by-voxel basis by calculating Kendall's coefficient of concordance for a given voxel time series with those of its nearest 26 neighbors. In our study, we used the ReHo method to examine altered brain regions and considered them as seeds for further functional connectivity analysis, which included the anterior cingulate cortex (ACC) and caudate putamen (CPu).

First, the images shot at the first 10 time points were eliminated, then the images were realigned in a time series. Those on which the head moved by more than 0.1 mm and 1 degree were excluded. The left qualified were registered to a template set which was based on a standard rat brain atlas of Paxinos and Watson. And the voxel of fMRI images was spatially normalized to 3 mm × 3 mm × 3 mm and smoothed with an isotropic Gaussian kernel (FWHM = 4 mm).

ReHo and functional connectivity analysis was operated on a Resting-State fMRI Data Analysis Toolkit V1.8 software (REST, http://www.restfmri.net). Multiple comparison corrections were conducted by family-wise error rate correction with a cluster-defining threshold (*P* = 0.001, two-tailed), relevant to a cluster level of *P* = 0.05.

### 2.5. Neurobehavioral Tests

Behavioral tests were performed as we previously described [25, 26].

All devices were provided by Shanghai Softmaze Information Technology Co. Ltd. (Shanghai, China). The rat behavior was videoed by an experienced investigator blind to the test.

#### 2.5.1. Open Field Test

An open field test was performed to evaluate the exploratory behavior and anxiety behavior.

The rats were centered into the black plastic chamber (100 *cm* × 100 *cm* × 40 *cm*) and allowed to explore it for 5 min. The total distance that a rat migrated and the time it took in the center of the open field were recorded by an experimenter blinded to the test. After one round of test, the arena was deodorized with 75% alcohol.

#### 2.5.2. Forced Swim Test

Rats were placed singly in a plastic cylinder (30 cm diameter and 80 cm height) filled with water (23-25°C) for 6 min, with the immobility scored in the final four minutes only. Time spent immobile (absence of movement except leg kicks to stay afloat) is then used as a measure of behavioral despair and helplessness, a rodent analogue of depressive-like behavior.

### 2.6. Enzyme-Linked Immunosorbent Assay (ELISA)

The rats were deep-anesthetized with 2% sodium pentobarbital in saline (60 mg/kg, intraperitoneally; Sigma Chemical Co., St. Louis, MO, USA). The blood was collected transcardially from thoracotomized rats and centrifuged at 4000 rpm for 5 min at 4°C. Plasma samples were stored at -80°C. The levels of interleukin-1*β* (IL-1*β*), tumor necrosis factor (TNF-*α*), IL-6, and brain-derived neurotrophic factor (BDNF) were measured by ELISA kits purchased from Jiancheng Biotechnology (Nanjing, China).

### 2.7. Statistical Analysis

Data presented as mean ± standard deviation (S.D.) was analyzed with SPSS 16.0. The Kolmogorov–Smirnov test was used for normally distributed data. Group comparisons were performed by two-sample *t*-tests. To determine the relationship between imaging data and depression-like behavior, Pearson's correlation was used. *P* < 0.05 was considered statistically significant.

## 3. Results

### 3.1. LPS Challenge Impaired Mood Function

In the open field test, LPS challenge resulted in a significantly decreased total distance traveled in the open field arena when compared with the control group (*t* = 11.048, *P* < 0.001, Figure 1(b)), suggesting that LPS induced reduced motility and sickness behavior. However, there was no difference in the time spent in the center arena of the open field between the two groups (*t* = 0.937, *P* = 0.357, Figure 1(c)). In the forced swimming test, immobility of the LPS group was significantly increased when compared with the control group (*t* = −4.354, *P* < 0.001, Figure 1(d)), indicating LPS challenge caused a depression-like behavior.

### 3.2. LPS Challenge Altered the ReHo Value

**Figure 2:**
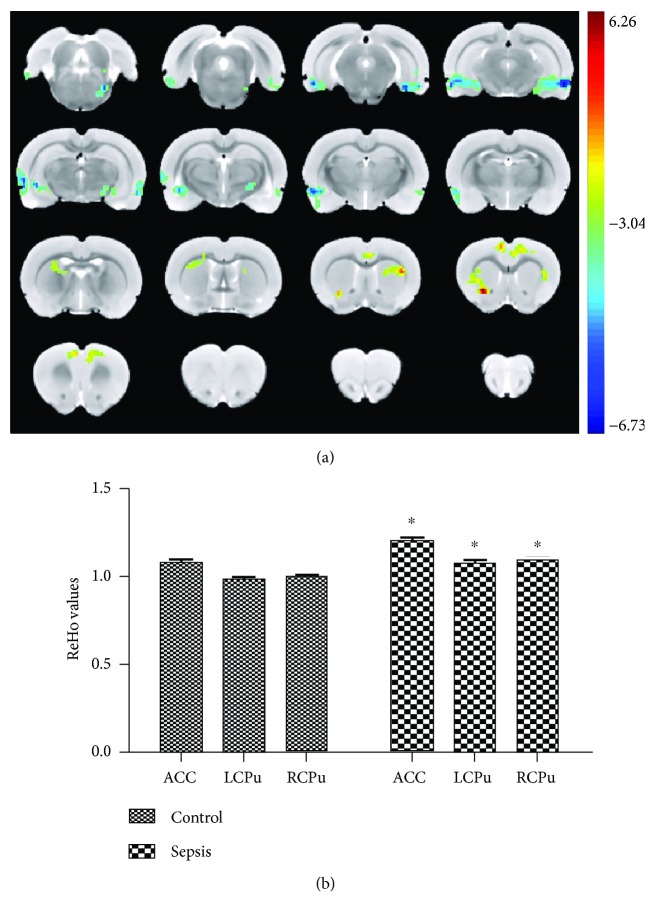
LPS challenge altered ReHo values. (a) The group difference distribution map of the ReHo (*P* < 0.05, corrected by family-wise error rate). Significantly increased ReHo are highlighted in the red-yellow heat bar scales; significantly decreased ReHo are highlighted in the blue-green heat bar scales. The ACC seed showed increased functional connectivity with the retrosplenial cortex compared with the control group. (b) The bar graph shows the mean ReHo values of ACC, LCPu, and RCPu with significant between-group differences extracted from the LPS and control groups. ACC: anterior cingulate cortex; LCPu: left caudate putamen; RCPu: right caudate putamen. ^∗^*P* < 0.05 vs. control group.

Since the main purpose of the study was to evaluate the influence of LPS challenge on cognitive function, we adopted the ReHo method, a data-driven analysis, after which the regions showing ReHo differences between the control and LPS groups were defined as seeds for further functional connectivity analysis. As shown in Figure 2, LPS challenge significantly increased ReHo values in rat brain regions, including the ACC and the CPu compared with the control group.

### 3.3. LPS Challenge Altered Functional Connectivity

**Figure 3:**
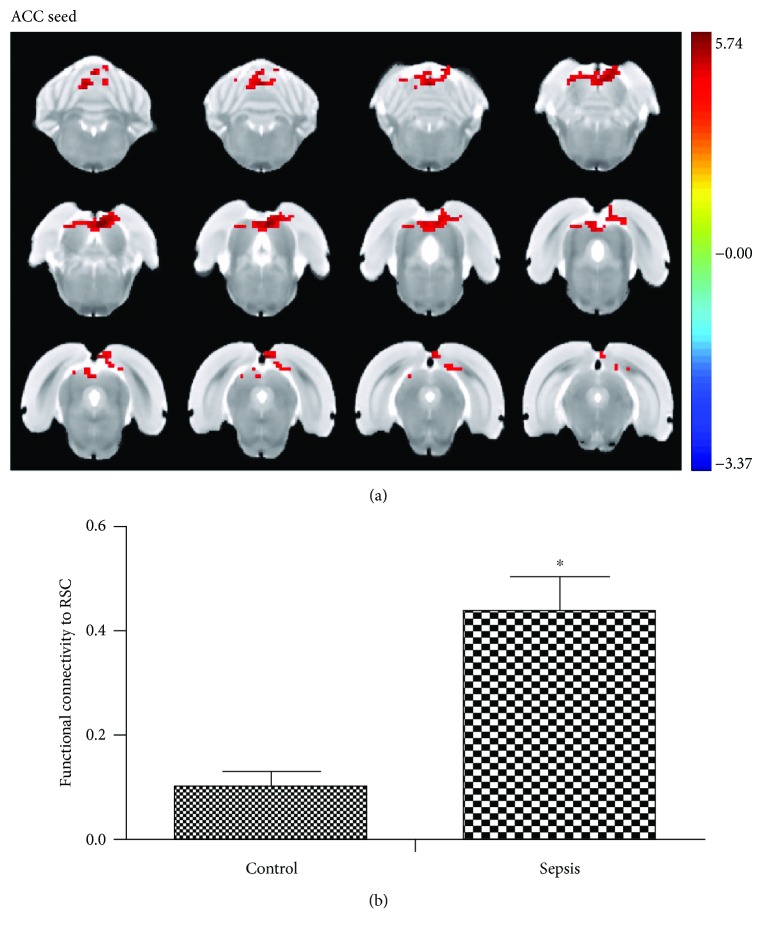
LPS challenge altered functional connectivity between the anterior cingulate cortex and retrosplenial cortex. (a) The group difference distribution map of the FC when the seed was set at the ACC (*P* < 0.05, corrected by family-wise error rate). Significantly increased FC are highlighted in the red-yellow heat bar scales. The ACC seed showed increased functional connectivity with the retrosplenial cortex compared with the control group. (b) The bar graph shows the mean FC *Z* values of RSC with the significant between-group difference extracted from the LPS and control groups. FC: functional connectivity, ACC: anterior cingulate cortex; RSC: retrosplenial cortex. ^∗^*P* < 0.05 vs. control group.

**Figure 4:**
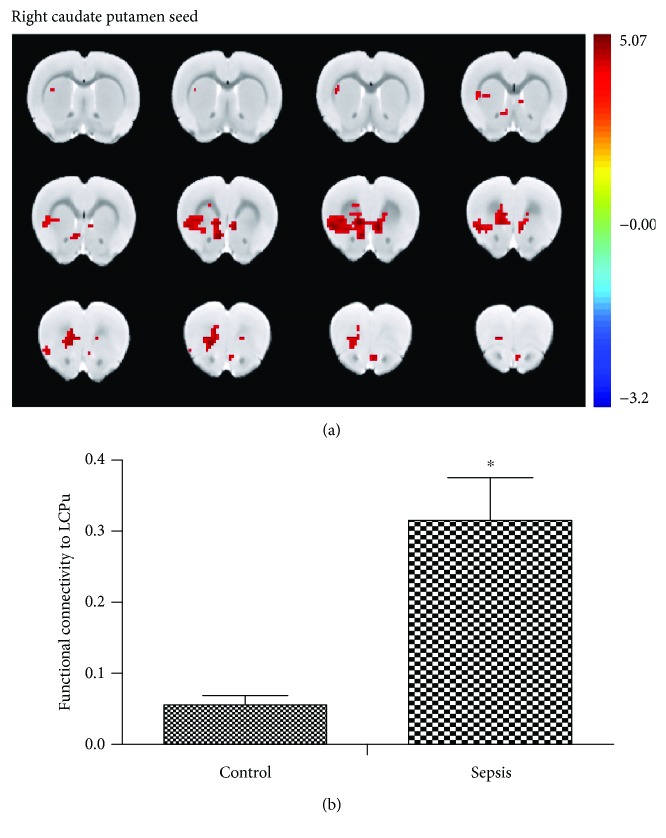
LPS challenge altered the functional connectivity between the right caudate putamen and left caudate putamen. (a) The group difference distribution map of the FC when the seed was set at the RCPu (*P* < 0.05, corrected by family-wise error rate). Significantly increased FC are highlighted in the red-yellow heat bar scales. The RCPu seed showed increased functional connectivity with the left CPu compared with the control group. (b) The bar graph shows the mean FC *Z* values of LCPu with the significant between-group difference extracted from the LPS and control groups. FC: functional connectivity; RCPu: right caudate putamen; LCPu: left caudate putamen. ^∗^*P* < 0.05 vs. control group.

After the analysis, we selected the ACC and the CPu as seeds of functional connectivity analysis. Compared with the control group, the ACC seed showed significantly increased functional connectivity with the retrosplenial cortex, superior colliculus, and inferior colliculus (Figure 3). In addition, the right CPu seed showed significantly increased functional connectivity with the left CPu (Figure 4).

### 3.4. LPS Challenge Did Not Change Plasma Proinflammatory Mediators but Decreased the BDNF Level

**Figure 5:**
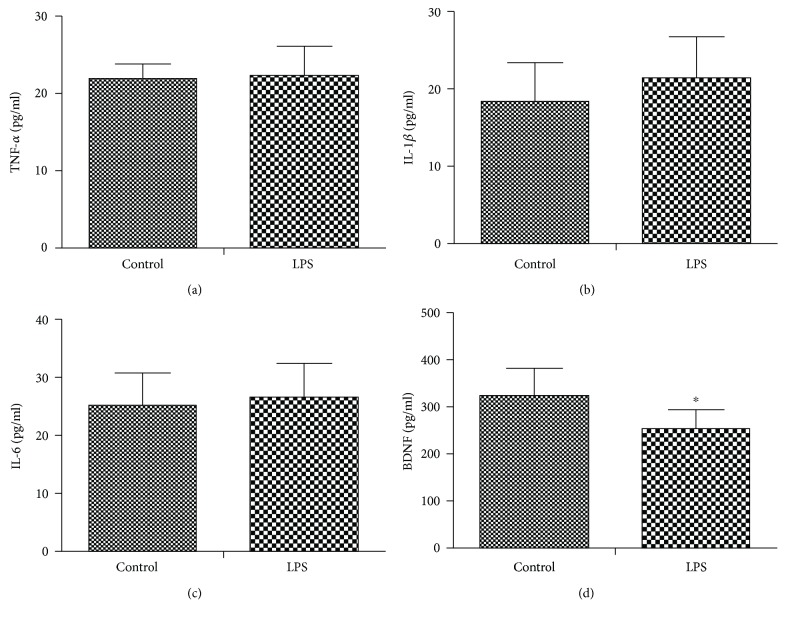
Effects of LPS challenge on plasma levels of proinflammatory mediators and BDNF. LPS challenge did not change the plasma proinflammatory mediators but decreased the BDNF levels. ^∗^*P* < 0.05 vs. control group.

As shown in Figures 5(a)–5(c), LPS challenge did not change plasma proinflammatory mediators when performed two days after injection compared with the control group (TNF-*α*: *t* = −0.334, *P* = 0.741, Figure 5(a); IL-1*β*: *t* = −0.1695, *P* = 0.101, Figure 5(b); IL-6: *t* = −0.673, *P* = 0.506, Figure 5(c)). However, our study showed that LPS challenge significantly decreased the plasma level of BDNF as compared with the control group (*t* = 3.783, *P* = 0.001, Figure 5(d)).

### 3.5. Correlation between Depression-Like Behavior and Neuroimaging Data

**Figure 6:**
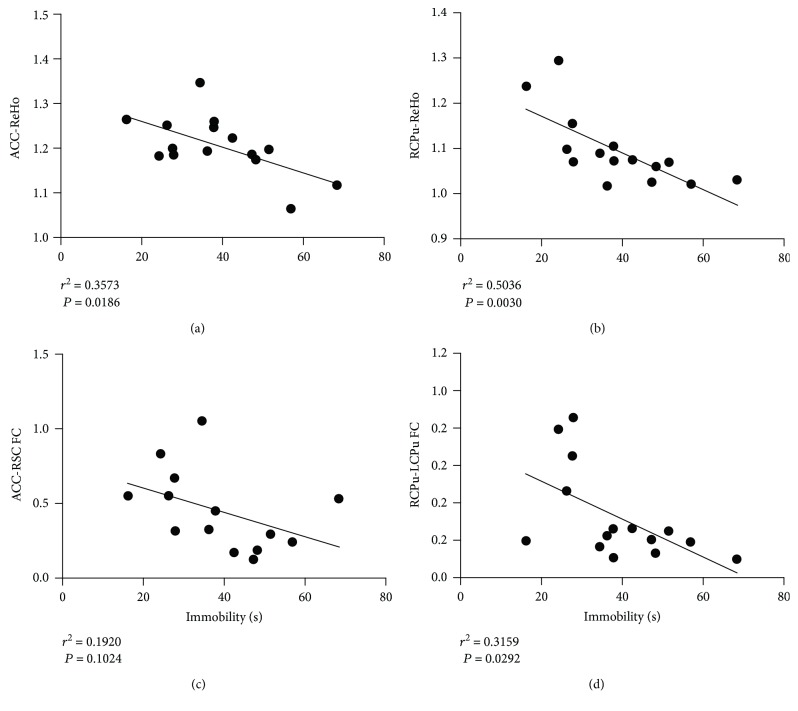
Correlation between depression-like behavior and neuroimaging data. The ReHo value in the two brain regions showed a correlation with depression-like behavior. The functional connectivity between the right CPu and the left CPu was significantly related to depression-like behavior. ACC: anterior cingulate cortex; LCPu: left caudate putamen; RCPu: right caudate putamen; RSC: retrosplenial cortex; FC: functional connectivity.

Within the LPS group, the ReHo values of the ACC and the right CPu showed significant correlations with the depression-like behavior (Figures 6(a) and 6(b)). Also, the functional connectivity between the right CPu and the left CPu was significantly related to depression-like behavior (Figure 6(d)). However, there was no difference in the functional connectivity between ACC and retrosplenial cortex and the depression-like behavior within the LPS group (Figure 6(c)).

### 3.6. Correlation between Plasma Level of BDNF and Neuroimaging Data

Neither abnormal ReHo brain regions nor functional connectivity between relevant brain regions was associated with plasma level of BDNF (data not shown).

## 4. Discussion

In the present study, we found that LPS induced significantly increased ReHo values in the ACC and the CPu and increased functional connectivity within the reward, motivation, and emotion regulation network. The abnormal spontaneous neuronal activity in those areas provided information on the neural mechanisms underlying cognitive impairment and suggested that ReHo analysis might be a useful noninvasive imaging tool for the detection of early cerebral abnormalities in sepsis patients. To the best of our knowledge, this is the first study examining the brain functional connectivity changes in a lipopolysaccharide- (LPS-) induced systemic inflammation animal model using BOLD-based fMRI based on ReHo analysis.

Systemic inflammation induces physiological and behavioral changes in both humans and animals and is involved in mood and cognitive impairments [1–3]. During neuroinflammation, elevated brain cytokine levels can mediate sickness behaviors and alter cognitive processes [3–5]. By using an acute immune challenge, our study suggested that LPS induced significantly impaired mood impairment as reflected by depression-like behavior performed in the forced swim test, which is consistent with prior studies [16–18]. Our study along with previous findings supports the notion that experimental endotoxin administration constitutes an animal model to study the pathophysiology of SAE [16, 22, 23]. However, the neural mechanisms by which systemic inflammation induced mood impairment remain to be elucidated.

Functional neuroimaging is a useful technique for exploring the neurophysiological mechanisms underlying various clinical disorders [7–9]. It has been demonstrated that altered temporal connectivity of the BOLD signal between brain regions that form networks are important for cognition [7–9]. Thus, analyzing functional connectivity may reveal spontaneous fluctuations in brain networks during resting state and thus allow mapping the intrinsic functional organization between spatially remote brain areas [27]. However, it is difficult to recognize precisely which regions are altered and drive the large-scale alterations of network synchronization. In contrast to functional connectivity, ReHo reflects the changes in temporal aspects of spontaneous neuronal activity in a brain region. It can be used to identify aberrant coherence of local neural activity across the entire brain [18]. The ReHo method has been proved useful in detecting the regional changes in a variety of neurological diseases, including amnesic mild cognitive impairment [28], depression [29], and hepatic encephalopathy [30]. However, no imaging study has thus far been performed to address the brain functional connectivity changes after peripheral LPS challenge based on ReHo analysis. Using this approach, we found that LPS challenge induced a significant increase in ReHo values in the ACC and the CPu. The finding of increased ReHo in these regions might indicate that there might exist functional abnormality, a phenomenon that can be explained by the recruitment of compensatory mechanisms. Thus, our study supports the notion of the functional impairment during systemic inflammation from a new perspective of regional brain spontaneous activity.

Most of the symptomatic effects of sepsis such as affective and cognitive impairment can be attributed to inflammation [2, 3]. However, our current study showed that LPS did not affect proinflammatory mediators when performed two days after LPS injection. One of the main reasons might be that the early cytokines such as TNF-*α*, IL-1*β*, and IL-6 peak shortly after LPS injection and return to the baseline levels within hours [16], providing one explanation for the negative results observed in our study. Indeed, a number of studies have shown that proinflammatory cytokines and anti-inflammatory cytokines return to normal after 24 hours after LPS stimulation [16, 31, 32]. Depression is one of the most common symptoms observed in sepsis patients [2, 3]. Brain regions involved in this neural circuit such as the CPu (an anatomy that is similar to the human corpus striatum), temporal lobe, amygdala, caudate, anterior cingulate cortex, and frontal cortex play a key role in the onset and/or maintenance of depression [33, 34]. Accordingly, we showed that the ReHo values in the ACC and the CPu increased significantly after LPS challenge, suggestive of increased synchronization of regional neural activity in these areas. Our findings of enhanced LPS-induced activation of the ACC and CPu are of interest because these regions have been reported to play a vital role in regulation of affective, interoceptive, and reward processing [35–37]. Consistently, it has been suggested that the mood-regulating circuit is greater in depressed animals compared with controls [11]. In addition, previous neuroimaging research has demonstrated that the ACC and the CPu are relevant brain regions in potential neural networks that are affected by peripheral inflammatory challenge [16, 38, 39]. This was further supported by the negative relationship between ReHo values in the ACC and CPu and the depression-like behavior. Thus, the ReHo of the ACC and CPu may serve as a potential biological marker for SAE.

Cognitive deficits reflect not only the alteration of specific brain regions but also disturbances of functional connectivity between different brain regions. One of the ways to estimate the brain function at a global level is to measure functional connectivity between brain regions [7]. Previous neuroimaging studies have demonstrated that systemic inflammation alters the functional connectivity of many brain networks at rest [16, 17]. Based on the ReHo analysis, we further suggested that LPS challenge induced significantly increased functional connectivity between the ACC and retrosplenial cortex. The involvement of the ACC and CPu in emotional processing and the interaction between emotion, reward, and cognition have been confirmed in a large number of studies [37–40]. Based on the previous cross-species cytoarchitectural mapping study, the rodent retrosplenial cortex can be related to the human precuneus [41], which is a key node of the default mode network (DMN). Given the functional regulation of the two higher-order brain structure, the ACC and retrosplenial cortex had been linked with negative emotional or behavioral symptoms. In addition, the abnormal alteration in functional connectivity between the ACC and retrosplenial cortex might potentially indicate disturbed DMN after LPS challenge. Thus, the impaired functionality of this region was correlated with depression-like symptoms. In support, an altered connectivity within key DMN regions is observed in patients with major depression [40]. On the other hand, the striatal circuitry has distinct involvement in behavioral responses regarding rewards and losses [42]. In support, our study showed increased right CPu and left CPu connectivity after LPS challenge, which might explain the disruption in emotion regulation and motivational behaviors in depression. Notably, the correlation of functional connectivity between the right CPu and the left CPu and the depression-like behavior may further support the important role of the disrupted network in SAE. However, it should be noted that some discrepancies existed between our study and previous findings; a phenomenon might be attributed to differences in species, LPS dose, and time to perform the fMRI [16, 17]. Together, our study that significantly increased ReHo value and functional connectivity in relevant brain regions suggested functional damage in SAE, but this speculation needs to be tested in future studies.

Some limitations should be mentioned in this study. Firstly, this is a cross-sectional study, which does not allow for causal conclusions about the relationship between alterations in functional connectivity and LPS-induced abnormal behaviors. Future longitudinal studies with anti-inflammatory intervention are needed to clarify this association. Secondly, it is difficult to perform these experiments on conscious animals due to motion artifacts; the inevitable influence of anesthetization on neural function cannot be excluded. Because some experimental manipulations are unsafe or unethical to perform in humans, such as larger-dose LPS challenge used in the present study, animal models are also necessary to improving our understanding of neurobiological processes that underlie systemic inflammation–induced mood and cognitive impairment. Finally, the relatively modest sample size may increase the risk of type II errors due to low statistical power.

To summarize, our study suggests that systemic inflammation induces mood impairment that is associated with abnormal regional spontaneous neuronal activity and resting-state functional network. Based on these data, this method may be used as an efficient way to assess inflammation effects on neurocircuitry in many psychiatric or medical illnesses associated with increased inflammation.

## Data Availability

The data used to support the findings of this study are available from the corresponding author upon request.
